# Dexamethasone for the prevention of a pain flare after palliative radiotherapy for painful bone metastases: a multicenter double-blind placebo-controlled randomized trial

**DOI:** 10.1186/1471-2407-14-347

**Published:** 2014-05-20

**Authors:** Paulien G Westhoff, Alexander de Graeff, Jenske I Geerling, Anna KL Reyners, Yvette M van der Linden

**Affiliations:** 1 Department of Radiotherapy, University Medical Center Utrecht, Q.00.118 Heidelberglaan 100, Utrecht 3584 CX, Netherlands; 2 Department of Medical Oncology, University Medical Center Utrecht, Utrecht, Netherlands; 3 Department of Medical Oncology, University of Groningen, University Medical Center Groningen, Groningen, Netherlands; 4 Department of Clinical Oncology, Leiden University Medical Center, Leiden, Netherlands

**Keywords:** Pain flare, Palliative radiotherapy, Dexamethasone, Bone metastases

## Abstract

**Background:**

Radiotherapy has a good effect in palliation of painful bone metastases, with a pain response rate of more than 60%. However, shortly after treatment, in approximately 40% of patients a temporary pain flare occurs, which is defined as a two-point increase of the worst pain score on an 11-point rating scale compared to baseline, without a decrease in analgesic intake, or a 25% increase in analgesic intake without a decrease in worst pain score, compared to baseline. A pain flare has a negative impact on daily functioning and mood of patients. It is thought to be caused by periostial edema after radiotherapy. Dexamethasone might diminish this edema and thereby reduce the incidence of pain flare. Two non-randomized studies suggest that dexamethasone reduces the incidence of a pain flare by 50%. The aim of this trial is to study the effectiveness of dexamethasone to prevent a pain flare after palliative radiotherapy for painful bone metastases and to determine the optimal dose schedule.

**Methods and design:**

This study is a three-armed, double-blind, placebo-controlled multicenter trial. We aim to include 411 patients with uncomplicated painful bone metastases from any type of primary solid tumor who receive short schedule radiotherapy (all conventional treatment schedules from one to six fractions). Arm 1 consists of daily placebo for four days, arm 2 starts with 8 mg dexamethasone before the (first) radiotherapy and three days placebo thereafter. Arm 3 consists of four days 8 mg dexamethasone. The primary endpoint is the occurrence of a pain flare. Secondary endpoints are pain, quality of life and side-effects of dexamethasone versus placebo. Patients complete a questionnaire (Brief Pain Inventory with two added questions about side-effects of medication, the EORTC QLQ-C15-PAL and QLQ-BM22 for quality of life) at baseline, daily for two weeks and lastly at four weeks.

**Discussion:**

This study will show whether dexamethasone is effective in preventing a pain flare after palliative radiotherapy for painful bone metastases and, if so, to determine the optimal dose.

**Trial registration:**

This study is registered at ClinicalTrials.gov: NCT01669499

## Background

Radiotherapy, with a single fraction of 8 Gray as the gold standard, has a good effect in palliation of painful bone metastases, with a pain response rate of more than 60%. [1] However, a possible side-effect is a transient progression of pain, the so-called pain flare. This pain flare is defined as a two-point increase of the worst pain score on an 11-point rating scale, compared to baseline, without a decrease in analgesic intake, or a 25% increase in analgesic intake without a decrease in worst pain score. A pain flare is distinguished from progression of pain by requiring the worst pain score and analgesic intake to return to baseline levels after the flare [2].

Two prospective observational studies show that approximately 40% of patients experience a pain flare [3,4]. Hird studied 111 patients with uncomplicated painful bone metastases and showed an incidence of pain flare of 40%, with no difference between single or multiple fractions. The median duration of a pain flare was 1.5 days, while 25% of patients had more than one pain flare. Most of the pain flares occurred during the first five days after treatment. A pain flare occurred in 52% of patients with breast cancer and in 25% of patients with prostate cancer [3]. Loblaw studied 44 patients and found, with an adjusted, underestimating definition of a pain flare, an incidence of 41%, with a significant difference between single and multiple fractions (57% and 24% respectively) [4].

A survey among patients who experienced a pain flare showed that having a pain flare had a negative effect on daily functioning of patients and on their mood [5]. Most patients tried to manage their pain flare by increasing their pain medication, at the cost of possible side-effects. The majority of patients who experienced a pain flare stressed the need for prevention of this pain flare instead of managing it with breakthrough medication.

The pain flare is thought to arise through edema of the periostium of the irradiated bone. Dexamethasone, an anti-inflammatory drug decreasing edema, may be an effective drug. Two small studies were performed to study the effect of dexamethasone on the incidence of pain flare [6,7]. In the study by Chow a single dose of 8 mg dexamethasone was prescribed one hour before the single fraction radiotherapy. This study included 33 patients and showed an overall pain flare incidence of 24%. Most of the observed pain flares commenced after the half-life of dexamethasone [7], suggesting that a longer treatment time might be useful. Dexamethasone was well tolerated. A subsequent study, with 41 evaluable patients, used 8 mg dexamethasone before single fraction radiotherapy and then daily for three consecutive days. It showed an overall incidence of pain flare of 22%, with a median duration of one day [6].

Both studies concluded that randomized trials are necessary to study the effectiveness of dexamethasone for prevention of a pain flare. No randomized trials have been published so far. Therefore, the aim of this trial is to study the effectiveness of dexamethasone to prevent a pain flare after palliative radiotherapy for painful bone metastases and to determine the optimal dose schedule.

## Methods/Design

### Design

**Figure 1:**
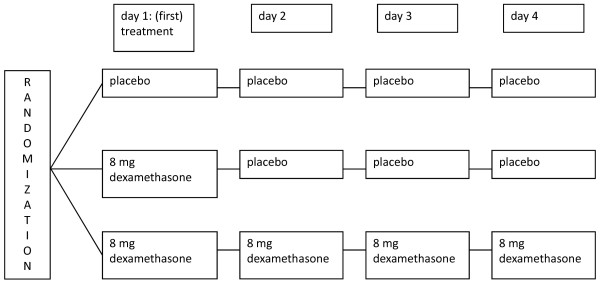
Treatment arms.

This three-armed, prospective, randomized, placebo-controlled multicenter study is being led by the University Medical Center Utrecht. The study is supported by grants from the Dutch Cancer Society and ZonMw. It is registered at ClinicalTrials.gov: NCT01669499. The study compares two different dose schedules of dexamethasone with a placebo (Figure 1). The aim is to study the effectiveness of dexamethasone to prevent the occurrence of a pain flare after radiotherapy for painful bone metastases and to define the optimal schedule of dosing. Secondary endpoints are pain scores, quality of life and side-effects of placebo and dexamethasone. In addition, the predictive value of a pain flare for response to the palliative radiotherapy will be studied.

### Patients

The study includes patients with uncomplicated painful bone metastases from a solid tumor, who are referred for a short course of palliative radiotherapy. Short course radiotherapy encompasses all conventional treatment schedules from one to six fractions of radiotherapy. The full inclusion and exclusion criteria are listed in Table 1. Patients are randomized between the three treatment arms (Figure 1), after stratification for center and treatment schedule: single or multiple fractions. Randomization is performed by telephone at the Comprehensive Cancer Center The Netherlands. Double-blind randomization is guaranteed by only communicating the number of the medication box to patients and physicians.

**Table 1:** Full inclusion and exclusion criteria

	
**Inclusion**	Patients of 18 years or older
	Uncomplicated painful bone metastases
	Primary malignancy is a solid tumor
	Pain intensity on a numeric rating scale of 2-8
	No immediately expected change in the analgesic regimen.
	Indication for single or short course radiotherapy
	Able to fill out Dutch questionnaires
	Able to follow instructions
	Informed consent provided
**Exclusion**	Patients with haematological malignancy
	Multiple sites to be irradiated
	Patients who have been treated before with palliative radiotherapy for painful bone metastases to the same bony localisation
	Current use of steroids (dexamethasone, prednisolone or other), use up to less than a week before randomization or expected use within 2 weeks after start of radiotherapy (e.g., as part of anti-emetic regimen for chemotherapy)
	Contraindications for the use of dexamethasone (to be judged by the radiation-oncologist)
	Long-term schedule radiotherapy (>6 fractions)
	Life expectancy shorter than 8 weeks
	Karnofsky performance score of 40 or less

### Participating centers

In total, 12 out of the 21 radiotherapy departments in the Netherlands participate in this nationwide study. Patients are asked for participation at these departments. Participating centers are:

University Medical Center Utrecht, Utrecht

Leiden University Medical Center, Leiden

MAASTRO Clinic, Maastricht

Medical Center Haaglanden, den Haag

The Netherlands Cancer Institute-Antoni van Leeuwenhoek Hospital, Amsterdam

Medical Spectrum Twente, Enschede

Erasmus Medical Center, Rotterdam

ARTI Institute for Radiation Oncology Arnhem, Arnhem

Institute Verbeeten, Tilburg

Catharina Hospital, Eindhoven

Zeeuws Radiotherapeutic Institute, Vlissingen

Reinier de Graaf Gasthuis, Delft

### Ethics, informed consent and safety

The protocol has been approved by the medical ethics committee of the University Medical Center Utrecht. In the participating centers, local medical ethics committees have approved the protocol. The study is conducted in accordance with the Declaration of Helsinki. Written informed consent, signed and dated, is obtained before randomization. Serious adverse events (SAE) or suspected unexpected serious adverse reactions (SUSAR), as defined in the study protocol, are reported to the central medical ethics committee and to the central committee of medical research involving human subjects.

### Endpoints and analysis

Participating patients fill out a questionnaire at baseline (the start of treatment, defined as day 1), then daily until day 15 and a final questionnaire at day 29. The questionnaire contains the Brief Pain Inventory [8], which notes the level of pain on a 11 point pain scale ranging from 0 (no pain) to 10 (worst imaginable pain). Two questions are added about side-effects of the study medication (‘Do you have appetite?’ and ‘Do you feel restless?’). To assess quality of life, the EORTC QLQ-C15-PAL [9] and the EORTC QLQ-BM22 [10] are added (both at baseline, day 8, 15 and 29). Reminder telephone calls are performed twice during the study. The researchers contact participants who do not return their questionnaires. The occurrence of a pain flare, the primary endpoint of the study, is determined using the daily noted pain scores and pain medication. Pain response and side-effects of placebo and dexamethasone are assessed by the daily questionnaires.

Analysis will be by intention-to-treat. Patients who have received at least one fraction of radiotherapy, irrespective of study medication intake, and have returned questionnaires are evaluable. Descriptive analyses of baseline characteristics will be performed. Comparison of occurrence of pain flare between the three arms will be assessed using the Chi-Square test. Comparison of pain intensity, quality of life items and side effects at baseline and over time between the three arms will be done using multilevel analysis. Since the risks of the study using well-known medication are considered minimal, an interim analysis will not be performed.

### Power calculation

Assuming a reduction of 50% (from 40 to 20%) of the occurrence of a pain flare by administering a single dose of 8 mg dexamethasone, a total of 411 patients are necessary (137 per arm) to reach a power of 90% given a significance level of 5% (2-sided), and assuming a drop-out of 20% during follow-up. In the Netherlands, around 3000 patients are eligible for this study yearly. With 12 out of 21 institutions participating and an estimated participation rate of 20%, the total study time needed is about 2 years.

## Discussion

Up to 40% of patients experience a pain flare after palliative radiotherapy for painful bone metastases [3,4]. A pain flare severely impacts functional activity and mood of patients [5]. Therefore, it is clinically important to prevent the occurrence of a pain flare. Earlier publications suggest an effect of dexamethasone on the incidence of pain flare [6,7]. Although side-effects of a short course and relative low dosage of dexamethasone are considered minimal, the beneficial effect in this patient population should be proven before integrating dexamethasone medication into daily clinical radiotherapy practice. A prolonged schedule of dexamethasone might be better in preventing a pain flare. Therefore, in the present trial, we study different schedules, to be able to determine which one is the optimum schedule.

A recent publication from Chiang et al. (published after the initiation of our study) in patients treated with stereotactic radiotherapy for painful bone metastases showed an incidence of pain flare of 68%. However, this might be an overestimation. Firstly, they did not mention to require pain score and analgesic intake to return to baseline, to distinguish it from progression. Secondly, initiation of corticosteroids during or after treatment was considered to be indicative of a pain flare [11]. Nevertheless, these results give rise to the assumption that this group of patients might also benefit from our treatment results. However, the results of our study are not directly applicable to patients who are treated with stereotactic radiotherapy for painful bone metastases, since they represent a highly selected group of patients who receive much higher total doses of radiotherapy (e.g. 1 × 20 Gy, or 3 × 8 Gy).

Using consensus definitions of endpoints in literature is important, to be able to compare studies [12]. Most published studies concerning pain flare after palliative radiotherapy use the definition by Chow [2], incorporating pain scores, analgesics intake and returning to baseline to distinguish it from progression. Loblaw et al. [4] used a different definition for pain flare. They tried to convert it into the definition by Chow [2], but since they used a different pain scale, this was not completely possible, which made it difficult to interpret and compare these results with other published studies. We chose to also use the definition by Chow [2], to enable comparison with other studies.

In conclusion, if this study proves the effectiveness of dexamethasone in the prevention of a pain flare after palliative radiotherapy for painful bone metastases, this should lead to a change in supportive care. Since we use a commonly accepted definition of pain flare, comparison between our results and future results from other trials may be possible. It may also lead to studies of the benefit of dexamethasone in preventing a pain flare after stereotactic radiotherapy.

## Abbreviations

BPI: Brief Pain Inventory; EORTC QLQ-C15-PAL: European Organisation for Research and Treatment of Cancer Quality of Life Questionnaire Core 15 for use in Palliative care; EORTC QLQ-BM22: European Organisation for Research and Treatment of Cancer Quality of Life Questionnaire for patients with Bone Metastases; SAE: Serious adverse event; SUSAR: Suspected unexpected serious adverse reaction.

## Competing interests

The authors declare that they have no competing interests.

## Authors’ contributions

The study protocol was drafted by AdG, YMvdL and AKLR. PGW and JIG participated in critical review of the study protocol. PGW is responsible for coordinating the data acquisition. This article was conceived and drafted by PGW, AdG and YMvdL. AKLR and JIG critically reviewed the manuscript. All authors have read and approved the final manuscript.

## Pre-publication history

The pre-publication history for this paper can be accessed here:

http://www.biomedcentral.com/1471-2407/14/347/prepub
